# Effect of defatted rice bran supplementation on metabolic parameters and inflammatory status in overweight/obese adults with hypercholesterolemia: a randomized, placebo-controlled intervention

**DOI:** 10.1186/s40795-022-00586-9

**Published:** 2022-09-01

**Authors:** Weeraya Saphyakhajorn, Rawiwan Sirirat, Suwimol Sapwarobol

**Affiliations:** 1 Department of Nutrition and Dietetics, Graduate Program in Food and Nutrition, Faculty of Allied Health Sciences, Chulalongkorn University, Bangkok, Thailand; 2 Center for Nutrition, Healthy Lifestyle, and Disease Prevention, School of Public Health, Loma Linda University, Loma Linda, California USA; 3 The Medical Food Research Group, Department of Nutrition and Dietetics, Faculty of Allied Health Sciences, Chulalongkorn University, Bangkok, Thailand

**Keywords:** Defatted rice bran, Hypercholesterolemia, Overweight, Obese

## Abstract

**Background:**

Defatted rice bran (DRB) is a byproduct of rice bran oil production rich in fiber, protein, and antioxidant compounds that may confer beneficial effects on metabolic profiles in humans. The current study aimed to investigate the effects of DRB supplementation on anthropometric and blood biochemical indices, dietary intake, and inflammatory status in overweight/obese subjects with hypercholesterolemia.

**Methods:**

In a 12-week-randomized placebo-controlled trial, 61 overweight/obese participants with a total cholesterol level > 200 mg/dL were randomly assigned either to 30 g/d DRB (*n* = 30) or to 10 g/d maltodextrin (*n* = 31).

**Results:**

DRB intervention significantly reduced systolic and diastolic blood pressure by 4.27 and 4.50%, respectively (126.20 ± 13.63 to 120.60 ± 13.72 mmHg, *p* = *0.0003* and 80.87 ± 7.38 to 77.17 ± 9.83 mmHg, *p* = *0.0035*). HbA1c also decreased significantly by 3.59% (5.89% ± 0.76% to 5.66% ± 0.62%, *p* = *0.0001*) after DRB supplementation. Total cholesterol, triglyceride, and low-density lipoprotein cholesterol levels also decreased insignificantly by 3.12, 1.32, and 1.53%, respectively, after DRB supplementation. Insignificant differences in fasting blood glucose, insulin, homeostatic model assessment of insulin resistance, quantitative insulin sensitivity check index, hs-CRP and homocysteine levels were also observed after DRB intervention. Reduction in caloric and fat intake were reported in DRB groups.

**Conclusions:**

DRB supplementation improved blood pressure and HbA1c levels. It also lowered blood cholesterol, albeit insignificantly. Caloric and fat intake were also significantly lower after DRB supplementation. Further study is needed to evaluate the mechanisms by which DRB improves these metabolic indices.

**Trial registration:**

Thai Clinical Trial Registration (https://www.thaiclinicaltrials.org/.) Thai Clinical Trial Registration number: TCTR20191020003. Registered 20 October 2019.

**Supplementary Information:**

The online version contains supplementary material available at 10.1186/s40795-022-00586-9.

## Background

Obesity is becoming an epidemic, with a prevalence rate that has tripled since 1975 [1]. In Thailand, the prevalence of obesity increased significantly from 33.9% in 2012 to 44.8% in 2018 [2]. Rice bran is a nutritious byproduct from rice milling that has been widely used for animal feed and rice bran oil production [3]. The process of rice bran oil extraction has long been established. This process produces not only rice bran oil but also defatted rice bran (DRB) as the main byproduct. A study on the physicochemical properties of DRB reported differences in the nutrient composition of DRB and full-fat rice bran to some extent. Even though some active ingredients (e.g., oryzanol, phytosterols, polyphenols, tocopherols, and tocotrienols) are excluded during the oil extraction process [4], DRB still holds a substantial amount of nutrients, including protein, non-starch polysaccharides, and antioxidant compounds [5, 6]. In addition, the protein digestibility of DRB is higher than that of full-fat rice bran [7]. These differences in nutrient composition and protein digestibility might alter the beneficial effects of DRB consumption in comparison to those of full-fat rice bran.

*In vitro* studies showed that rice protein hydrolysate (RPH) lowers blood pressure by inhibiting angiotensin-converting enzyme (ACE) and renin activities [8, 9]. In animal studies, DRB also demonstrated an antihypertensive effect by inhibiting ACE activity and increasing nitric oxide (NO) bioavailability. In a study on rats, phytochemical compounds in DRB exerted an anti-inflammatory effect [10, 11]. Additionally, DRB demonstrated antioxidant activity in an animal model by reducing plasma malondialdehyde and superoxide production and suppressed p47phox NADPH oxidase expression in rats fed with a high-carbohydrate and high-fat diet [10]. Antidiabetic and anticholesterolemic effects were also observed in animal studies [12, 13].

Currently, research on the effects of DRB supplementation on metabolic parameters in humans is limited. In light of this inadequate information, DRB is currently used only as animal feed. Proof of its effect on metabolic indices may provide justification for the use of DRB as an active ingredient in functional foods. This study, therefore, aimed to investigate the effects of DRB supplementation on body weight, lipid profiles, metabolic parameters, and inflammatory status in overweight/obese adults with hypercholesterolemia.

## Methods

### Preparation of defatted rice bran

A mixture of local brown Thai rice (*Oryza sativa L.*) varieties were procured from a local rice mill in central Thailand. Full-fat rice bran was obtained after the milling process and stabilized by heat treatment prior to oil extraction. In the solvent extraction process, stabilized rice bran was extracted with n-hexane. This procedure yielded crude rice bran oil and DRB. The crude rice bran oil contained 41.13% monounsaturated fatty acids (40.6% oleic acids), 34.24% polyunsaturated fatty acids (32.92% linoleic acids), and 24.63% saturated fatty acids (20.9% palmitic acids) (Gas Chromatography AOCS 1c-89). Rice bran was heated to 120 °C–130 °C for 30 s via steam and high compression friction. DRB was powdered, heated to reduce the moisture content to less than 6%, passed through a 60-mesh sieve, and stored in airtight containers under hygienic conditions at room temperature in a dry place until further use. These processes were performed at the Thai Ruam Jai Vegetable Oil Co., Ltd. Thailand.

In this clinical trial, DRB was obtained in one batch to maintain homogeneity. For safety purposes, microorganisms (*Escherichia coli, Staphylococcus aureus*, and total coliforms), and other toxic substances (Lead, Cadmium, Arsenic, Alflatoxin) were tested, and the results showed values within the normal range according to the guidelines of the Thai Food and Drug Administration. The protein (amino acids) and fat contents and micro-nutrient composition were determined according to the AOAC standard protocol [14]. Before the clinical trial, 15 g of DRB was weighed and tightly sealed in an aluminum sachet. Five g of tapioca-maltodextrin was packed in the same size and type of aluminum sachet to be used as a placebo control. Maltodextrin was purchased from Krungthepchemi, Bangkok, Thailand. The nutritional composition of DRB (30 g) and Maltodextrin (10 g) is shown in Table 1. In this study, 30 g of DRB provided 90 kcal, 17.78 g carbohydrates, 5.55 g protein, 7.78 g fiber, and 0 g fat. Maltodextrin 10 g provided 40 kcal and 9.5 g carbohydrates.

**Table 1:** The nutritional composition of DRB (30 g) and maltodextrin (10 g)

**Nutrients**	**DRB** **(30 g)**	**Maltodextrin** **(10 g)**
Energy (kcal)	90	40
Carbohydrates (g)	17.78	9.5
Protein (g)	5.55	0
Fat (g)	0	0
Fiber (g)	7.78	0

### Study design

Participants were recruited using a poster advertisement in the neighborhood of Chulalongkorn University, Bangkok, Thailand. A nurse and a registered dietitian screened participants for the inclusion criteria, which included age 18–60 years, overweight or obese, body mass index (BMI) ≥ 23 kg/m^2^, fasting total cholesterol (TC) > 200 mg/dL, and no known metabolic-related diseases, rice bran allergies, or eating disorders. Participants who smoked, drank alcoholic beverages, had any metabolic disorders, and/or took any medication and dietary supplements related to weight control or that could have confounded any study indicators were excluded.

**Fig. 1:**
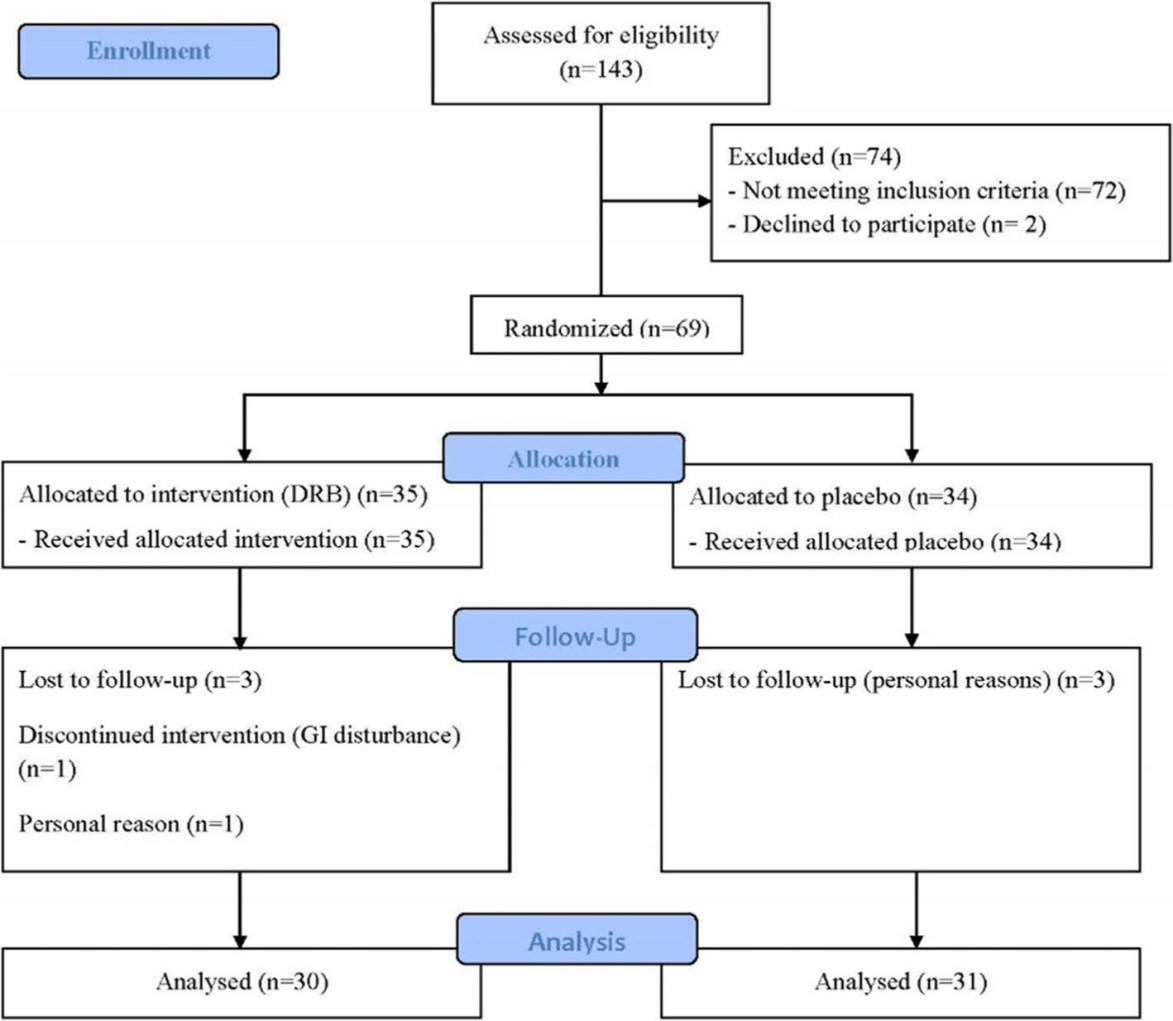
CONSORT flow diagram of the study

A 12-week, double-blinded, randomized controlled trial was conducted to examine the metabolic properties of DRB in overweight/obese participants with hypercholesterolemia. Sixty-nine participants complied with the inclusion criteria and were randomly allocated (according to www.graphpad.com) to one of the following groups: the intervention (DRB) group (*n* = 35) or the placebo control group (*n* = 34). In the DRB group, five participants were withdrawn from the study: three were lost to follow up, one had GI disturbance, and the other withdrew for personal reasons. In the control group, three participants were lost to follow up. In total, 31 participants (23 females, 8 males) in the control group and 30 participants (21 females, 9 males) in the DRB group completed this study (Fig. 1).

Participants were advised to consume two sachets of DRB (15 g DRB per sachet) or two sachets of placebo (5 g maltodextrin per sachet) daily before regular meals (breakfast and dinner). During the 12 weeks of the intervention, participants were requested to continue their usual diets and maintain their usual levels of physical activity throughout the study. In addition, they were instructed not to consume any other rice bran or rice bran-derived products during the study.

After a week-long run-in period, both groups of participants were requested to visit the clinic at the Department of Nutrition and Dietetics, Chulalongkorn University, Bangkok, Thailand, five times—at weeks 0 (baseline), 3, 6, 9, and 12 after the intervention—to examine the parameters of interest, including blood pressure, anthropometric parameters, and dietary records. Venous blood was drawn at weeks 0, 6, and 12 for measurement of the parameters of interest, including fasting blood glucose (FBG), insulin, HbA1c, fasting blood lipid profiles (TC, TG, HDL-c, and LDL-c), inflammatory cytokines (hs-CRP), and homocysteine levels. At each clinic visit, a three week's supply of the test substance was distributed, any unused sachets from the previous visit were collected and counted. The participants were followed up for compliance by random phone calls twice weekly (one weekday and one weekend day).

### Anthropometric assessment

Body weight, muscle mass, fat mass, and fat-free mass were measured using a bioelectrical impedance analyzer (MC-980 MA body composition analyzer, TANITA Corporation, Tokyo, Japan). Participants were dressed in light attire and barefoot. Eight polar electrodes were positioned such that an electric current was supplied from the electrodes on both feet and hands. Voltage was then measured on the heels of both feet and the near sides of both hands. Waist circumference was measured to the nearest 1.0 cm using a standard measuring tape at a point immediately above the iliac crest on the mid-axillary line at minimal respiration. BMI was calculated as weight/height^2^ (in kg/m^2^). Blood pressure was measured using an OMRON HEM-8712 blood pressure monitor. Participants were advised to remain seated and relaxed for five minutes before the measurement with their legs uncrossed and their back supported. Blood pressure measurement was duplicated with a 5-min interval, and the average value was recorded [15].

The visceral adiposity index (VAI) was calculated as described [16] using the following sex-specific equations, where TG is the triglyceride level, expressed in mmol/l, and HDL is the HDL-cholesterol level, expressed in mmol/l:

$$\mathrm{Female}\;\mathrm{VAI}=\left(\frac{\mathrm{Waist}\;\mathrm{circumference}\;(\mathrm{cm})}{36.58+(1.89\times\mathrm{BMI})}\right)\times\left(\frac{\mathrm{TG}}{0.81}\right)\times\left(\frac{1.52}{\mathrm{HDL}}\right)$$

$$\mathrm{Male}\;\mathrm{VAI}=\left(\frac{\mathrm{Waist}\;\mathrm{circumference}\;(\mathrm{cm})}{39.68+(1.88\times\mathrm{BMI})}\right)\times\left(\frac{\mathrm{TG}}{1.03}\right)\times\left(\frac{1.31}{\mathrm{HDL}}\right)$$

Relative fat mass (RFM) was calculated by using the following equation:

$$\mathrm{RFM}=64- \left(20 \times \frac{\mathrm{height }\left(\mathrm{m}\right)}{\mathrm{waist }\left(\mathrm{m}\right)}\right) + \left(12 \times \mathrm{sex}\right)$$

where height and waist circumference are expressed in meters. Sex = 0 for male and 1 for female [17].

### Blood biochemical assessment

At each clinic visit, a medical technologist or nurse drew a blood sample of approximately 15 ml by vein puncture after an overnight fast of 10–12 h. After collection, blood samples were separated into four tubes. For fasting glucose concentration determination, blood samples were kept in sodium-fluoride tubes. For %HbA1c and homocysteine determination, samples were kept in EDTA tubes. In addition, for fasting lipid, insulin and hs-CRP determination, blood samples were kept in two tubes with clot activator.

Blood glucose was examined by the hexokinase method using a clinical chemistry analyzer (Beckman Coulter AU480, USA), whereas TC, LDL-c, HDL-c, and TG were examined using the enzymatic method (Beckman Coulter, USA). Serum insulin levels were analyzed by the chemiluminescence immunoassay method (CLIA) [18]. Blood samples were immediately centrifuged (3,000 rpm) for 10 min at 4 °C and examined on the day of blood collection. For serum hs-CRP and homocysteine analysis, blood samples were immediately centrifuged (3,000 rpm) for 10 min at 4 °C, and the specimens were kept at − 80 °C for further analysis. Serum hs-CRP was measured by turbidimetric immunoinhibition assay (Beckman Coulter, USA). Serum homocysteine was analyzed by the chemiluminescence immunoassay method (Abbott Diagnostics).

All metabolic outcomes were examined at the Health Sciences service unit, Faculty of Allied Health Sciences, Chulalongkorn University. Additionally, the homeostatic model assessment of insulin resistance (HOMA-IR) was calculated as fasting serum insulin (μIU/mL) × fasting plasma glucose (mg/dL)/405. A quantitative insulin sensitivity check index (QUICKI) was calculated as a log transformation of the insulin glucose product. QUICKI = 1/[log(fasting insulin) + log(fasting glucose)] [19, 20].

### Dietary intake assessment

A weekly (two weekdays and one weekend) diet record was collected and examined for average intakes throughout the 12 weeks of the intervention period. Energy and macronutrient intake was calculated by using the food composition database in INMUCAL Nutrients software version 3 (developed by the Institute of Nutrition, Mahidol University, Thailand), which is based on Thai food composition and recipes [21]. The average daily intake of energy, carbohydrates, protein, fat, and dietary fiber of the DRB and placebo groups were presented as an average of energy and nutrients recorded in the week prior to the study (which represents the baseline data), as well as during the study.

### Gastrointestinal symptom assessment

Participants were instructed to record their gastrointestinal symptoms, including flatulence, borborygmi, nausea, vomiting, stomach pain, and passing flatus by means of a gastrointestinal symptom questionnaire. Participants rated the intensity of symptoms as 0 (none), 1 (mild), 2 (moderate), or 3 (severe). A total score was calculated for the intensity of all symptoms. Participants also evaluated their stool form by using the Bristol stool scale with a picture and description for each type of stool form [22].

### Statistical analysis

The sample size was calculated based on the difference in the serum total cholesterol between the groups from the previous study of Hongu et al. [23], and the power and alpha levels set at 80% and at 0.05, respectively. A sample size of 29 participants (in each group) was considered adequate. Statistical analyses were conducted using SPSS software for Windows (version 22.0; SPSS, Inc., Chicago, IL). The normal distribution of the values was checked by a Kolmogorov–Smirnov test. Continuous variables were presented as the means and standard deviations, while categorical data were presented as numbers and percentages. The categorical variables were compared with a chi-square test. An independent t-test was used to compare continuous variables at the beginning of the study and the mean changes in these variables during the intervention between the two groups. To analyze group changes at the baseline and follow-up weeks, a repeat-measured ANOVA was used. Tukey’s multiple comparison test was used to compare the groups when ANOVA test results were significant. All statistical analyses were 2-sided and evaluated at *p* = 0.05.

## Results

At baseline, there was no significant difference in anthropometric, blood biochemical, and dietary intake parameters between the placebo (*n* = 31, 23 females, 8 males) and DRB (*n* = 30, 21 females, 9 males) groups. However, HDL-c at baseline was significantly higher in DRB participants (57.7 ± 13.21 mg/dL) than in the placebo group (51.35 ± 10.21 mg/dL) *p* = *0.0397* (Table 2).

**Table 2:** Baseline characteristics of the placebo (*n* = 31) and DRB (*n* = 30) groups

**Parameters**	**Placebo (*n* = 31)**	**DRB (*n* = 30)**
**Anthropometrics parameters**		
Age (years)	31.71 ± 12.27	36.87 ± 12.30
Sex		
Female	23 (74.2%)	21 (70.0%)
Male	8 (25.8%)	9 (30.0%)
Height (cm)	163.48 ± 9.16	162.08 ± 8.26
Body weight (kg)	75.38 ± 15.56	77.76 ± 16.75
BMI (kg/m^2^)	28.10 ± 4.50	29.45 ± 4.57
Waist circumference (cm)	93.60 ± 11.03	95.09 ± 10.86
Fat mass (kg)	27.68 ± 10.26	30.21 ± 10.20
Fat-free mass (kg)	47.74 ± 10.82	47.48 ± 10.43
Muscle mass (kg)	45.02 ± 10.38	44.52 ± 10.48
Relative fat mass	37.60 ± 6.63	38.30 ± 5.58
Visceral adiposity index	2.07 ± 1.00	1.64 ± 0.96
SBP (mmHg)	122.13 ± 15.05	126.20 ± 13.63
DBP (mmHg)	78.45 ± 10.32	80.87 ± 7.38
**Blood biochemical parameters**		
FBG (mg/dL)	99.13 ± 27.95	94.93 ± 22.79
HbA1c (%)	5.89 ± 0.67	5.89 ± 0.76
Serum Insulin (uIU/mL)	9.16 ± 4.28	8.50 ± 4.37
HOMA-IR	2.10 ± 1.04	2.14 ± 1.50
QUICKI	0.35 ± 0.03	0.35 ± 0.03
TC (mg/dL)	236.32 ± 30.44	242.00 ± 46.45
TG (mg/dL)	131.27 ± 58.99	121.52 ± 64.94
LDL-c (mg/dL)	158.94 ± 33.57	165.40 ± 37.69
HDL-c (mg/dL)	51.35 ± 10.21	57.7 ± 13.21*
LDL:HDL ratio	3.19 ± 0.80	3.06 ± 0.87
hs-CRP (mg/L)	2.74 ± 1.91	1.88 ± 1.59
Homocysteine (μmol/L)	10.69 ± 3.07	11.37 ± 3.29
**Dietary intake**		
Energy (kcal/day)	1,689.29 ± 428.64	1,770.4 ± 257.16
Carbohydrate (g/day)	212.29 ± 57.58	225.37 ± 45.77
Protein (g/day)	74.09 ± 31.16	71.94 ± 15.24
Fat (g/day)	60.81 ± 20.72	64.80 ± 16.39
Energy distribution		
Carbohydrate (%)	51.35 ± 7.03	51.35 ± 7.53
Protein (%)	17.10 ± 4.58	16.37 ± 2.86
Fat (%)	31.55 ± 6.04	32.28 ± 6.37
**Gastrointestinal symptoms parameters**		
Flatulence	0.4 ± 0.82	0.41 ± 0.68
Borborygmi	0.63 ± 0.72	0.59 ± 0.82
Nausea	0.07 ± 0.25	0.10 ± 0.56
Vomiting	0.00 ± 0.00	0.07 ± 0.37
Stomach pain	0.27 ± 0.69	0.17 ± 0.47
Passing flatus	0.73 ± 0.91	0.66 ± 0.81
Bristol stool form	4.37 ± 1.38	4.17 ± 1.23

All values are expressed as the mean ± SD. Significant differences between categorical variables of the two study groups were determined by the chi-square test. Significant differences between continuous variables of the two study groups were determined by independent t-tests. *P-value ≤ 0.05 is considered to indicate a statistically significant result

*SBP* Systolic blood pressure, *DBP* Diastolic blood pressure, *BMI* Body mass index, *FBG* Fasting blood glucose, *HOMA-IR* The homeostatic model assessment of insulin resistance, *QUICKI* The quantitative insulin sensitivity check index, *TC* Total cholesterol, *TG* Triglycerides, *LDL-c* Low-density lipoprotein, *HDL-c* High-density lipoprotein

### Anthropometric parameters

The study did not show any significant differences in body weight between the DRB and placebo groups after the 12-week intervention: (77.76 ± 16.75 kg to 77.99 ± 16.51 kg and 75.38 ± 15.56 kg to 75.28 ± 15.29 kg, respectively). Likewise, no significant alterations in the remaining body composition parameters between groups were revealed (Table 3). However, systolic blood pressure was significantly decreased by 4.27% after 12 weeks of DRB supplementation (126.20 ± 13.63 to 120.60 ± 13.72 mmHg, *p* = *0.0003*). Moreover, the diastolic blood pressure of participants supplemented with DRB decreased significantly by 4.50% after intervention compared to baseline (80.87 ± 7.38 vs. 77.17 ± 9.83 mmHg, *p* = *0.0035*), while there were no significant changes in blood pressure in the placebo group.

**Table 3:** Comparison of anthropometric, blood biochemical, and dietary intake parameters of the placebo (*n* = 31) and DRB (*n* = 30) groups

Parameters	Placebo (*n* = 31)			Mean change	DRB (*n* = 30)			Mean change
	Baseline	Week 6	Week 12		Baseline	Week 6	Week 12	
**Anthropometric parameters**								
Body weight (kg)	75.38 ± 15.56	75.3 ± 15.63	75.28 ± 15.29	− 0.10 ± 1.80	77.76 ± 16.75	78.00 ± 16.60	77.99 ± 16.51	0.20 ± 1.39
BMI (kg/m^2^)	28.10 ± 4.50	28.12 ± 4.56	28.13 ± 4.56	0.03 ± 0.71	29.45 ± 4.57	29.52 ± 4.64	29.55 ± 4.66	0.06 ± 0.63
Waist circumference (cm)	93.60 ± 11.03	93.31 ± 11.24	93.44 ± 11.27	− 0.16 ± 1.19	95.09 ± 10.86	95.40 ± 11.06	95.28 ± 10.81	0.15 ± 0.93
Fat mass (kg)	27.68 ± 10.26	27.59 ± 10.54	27.43 ± 10.03	− 0.25 ± 1.68	30.21 ± 10.20	30.26 ± 10.52	30.46 ± 10.26	0.23 ± 1.33
Fat-free mass (kg)	47.74 ± 10.82	47.82 ± 10.70	47.69 ± 10.99	− 0.05 ± 1.16	47.48 ± 10.43	47.48 ± 10.25	47.60 ± 10.41	0.10 ± 1.09
Muscle mass (kg)	45.02 ± 10.38	45.10 ± 10.25	45.03 ± 10.62	0.02 ± 1.09	44.52 ± 10.48	44.90 ± 9.94	44.93 ± 10.02	0.37 ± 2.26
Relative fat mass	37.60 ± 6.63	37.47 ± 6.66	37.52 ± 6.62	− 0.08 ± 0.49	38.30 ± 5.58	38.39 ± 5.69	38.37 ± 5.68	0.05 ± 0.31
Visceral adiposity index	2.07 ± 1.00	2.07 ± 0.96	2.01 ± 0.99	− 0.04 ± 0.69	1.64 ± 0.96	1.50 ± 0.67	1.54 ± 0.93	− 0.13 ± 0.77
SBP (mmHg)	122.13 ± 15.05	121.32 ± 14.60	123.52 ± 13.93	1.39 ± 9.67	126.20 ± 13.63^a^	123.33 ± 13.07^a,b^	120.60 ± 13.72^b^	− 5.6 ± 8.37
DBP (mmHg)	78.45 ± 10.32	80.00 ± 8.19	79.19 ± 8.81	0.74 ± 7.13	80.87 ± 7.38^a^	77.40 ± 10.89^b^	77.17 ± 9.83^c^	− 3.7 ± 7.50
**Blood biochemical parameters**								
FBG (mg/dL)	99.13 ± 27.95	100.35 ± 30.88	101.19 ± 31.79	2.06 ± 9.27	94.93 ± 22.79	96.60 ± 21.98	96.57 ± 22.40	1.63 ± 6.88
HbA1c (%)	5.89 ± 0.67^a^	5.81 ± 0.79^a,b^	5.78 ± 0.69^b^	− 0.11 ± 0.18	5.89 ± 0.76^a^	5.77 ± 0.70^b^	5.66 ± 0.62^c^	− 0.23 ± 0.28
Insulin (uIU/mL)	9.16 ± 4.28	8.86 ± 4.59	8.99 ± 5.04	− 0.35 ± 2.87	8.50 ± 4.37	8.25 ± 3.26	8.38 ± 3.88	− 0.13 ± 2.40
HOMA-IR	2.10 ± 1.04	2.06 ± 1.08	2.10 ± 1.29	0.07 ± 0.77	2.14 ± 1.50	2.13 ± 1.46	2.15 ± 1.53	0.01 ± 0.58
QUICKI	0.35 ± 0.03	0.35 ± 0.04	0.35 ± 0.04	0.00 ± 0.02	0.35 ± 0.03	0.35 ± 0.04	0.35 ± 0.03	− 0.00 ± 0.02
TC (mg/dL)	236.32 ± 30.44	230.94 ± 34.40	238.03 ± 36.59	1.71 ± 20.03	246.40 ± 45.22	242.00 ± 46.45	238.27 ± 47.31	− 8.13 ± 22.43
TG (mg/dL)	131.27 ± 58.99	133.4 ± 59.42	130.10 ± 53.52	2.00 ± 43.71	121.52 ± 64.94	112 ± 46.87	112.24 ± 54.46	− 11.07 ± 56.02
LDL (mg/dL)	158.94 ± 33.57^a^	164.58 ± 36.83^a,b^	169.58 ± 35.11^b^	10.65 ± 22.55	168.73 ± 37.59	165.40 ± 37.69	166.27 ± 41.54	− 2.47 ± 16.95
HDL (mg/dL)	51.35 ± 10.21	52.35 ± 11.10	53.23 ± 11.69	1.87 ± 6.32	57.7 ± 13.21	56.97 ± 14.42	57.63 ± 14.73	− 0.07 ± 7.05
LDL:HDL ratio	3.19 ± 0.80	3.25 ± 0.84	3.32 ± 0.93	0.14 ± 0.38	3.06 ± 0.87	3.04 ± 0.87	3.02 ± 0.86	− 0.04 ± 0.43
hs-CRP (mg/L)	2.74 ± 1.91	3.11 ± 3.30	2.79 ± 2.35	− 0.55 ± 3.21	1.88 ± 1.59	2.28 ± 1.83	2.09 ± 1.94	0.22 ± 1.35
Homocysteine (μmol/L)	10.69 ± 3.07	10.37 ± 2.87	11.06 ± 2.46	0.36 ± 2.99	11.37 ± 3.29	11.10 ± 3.12	10.98 ± 3.20	− 0.39 ± 2.30
**Dietary intake parameters**								
Energy (kcal/day)	1,689.29 ± 428.64	1,715.08 ± 460.73	1,777.30 ± 444.95	88.01 ± 949.19	1,770.40 ± 257.16^a^	1,723.56 ± 374.34^a,b^	1,646.16 ± 339.87^b^	− 124.24 ± 381.77
Carbohydrate (g/day)	212.29 ± 57.58	230.65 ± 77.81	233.93 ± 178.47	21.64 ± 184.05	225.37 ± 45.77	226.04 ± 42.55	216.55 ± 51.27	− 8.83 ± 58.16
Protein (g/day)	74.09 ± 31.16	69.65 ± 19.11	72.00 ± 32.94	− 2.09 ± 23.98	71.94 ± 15.24	72.54 ± 17.62	71.45 ± 14.28	− 0.49 ± 21.40
Fat (g/day)	60.81 ± 20.72	56.72 ± 18.77	60.86 ± 26.05	0.05 ± 25.33	64.80 ± 16.39^a^	58.84 ± 22.20^a,b^	59.62 ± 21.88^b^	− 9.77 ± 19.62
Energy distribution								
Carbohydrate (%)	51.35 ± 7.03	53.12 ± 7.22	55.22 ± 28.18	3.87 ± 29.82	51.35 ± 7.53	51.63 ± 6.31	51.08 ± 5.47	− 0.27 ± 7.27
Protein (%)	17.10 ± 4.58	16.77 ± 3.06	17.99 ± 5.07	0.88 ± 4.90	16.37 ± 2.86	16.55 ± 2.47	17.34 ± 2.75	0.97 ± 3.70
Fat (%)	31.55 ± 6.04	30.11 ± 6.06	33.46 ± 8.33	1.92 ± 9.24	32.28 ± 6.37	31.82 ± 6.26	31.58 ± 6.06	− 0.70 ± 6.85
Dietary fiber (g/day)	6.98 ± 3.28	6.25 ± 2.88	7.47 ± 5.91	0.49 ± 5.57	9.48 ± 5.35^a^	15.40 ± 2.99^b^	15.38 ± 3.33^c^*	5.90 ± 5.45

All values are expressed as the mean ± SD. Significant differences at each follow-up time within a group were determined by repeated measures ANOVA. Significant difference between two study groups were determined by independent t-tests. Different letters in the same row refer to significant differences at each follow-up week. *refers to significant differences between study groups at week 12. A *P*-value ≤ 0.05 is considered statistically significant for all tests

*BMI* Body mass index, *SBP* Systolic blood pressure, *DBP* Diastolic blood pressure, *FBG* Fasting blood glucose, *HOMA-IR* The homeostatic model assessment of insulin resistance, *QUICKI* The quantitative insulin sensitivity check index, *TC* Total cholesterol, *TG* Triglycerides, *LDL* Low-density lipoprotein, *HDL* High-density lipoprotein, *hs-CRP* High sensitivity C-reactive protein

### Blood biochemical parameters

Total cholesterol, TG, and LDL-c levels decreased insignificantly by 3.12, 1.32, and 1.53% after DRB supplementation (246.40 ± 45.22 to 238.27 ± 47.31 mg/dL, 121.52 ± 64.94 to 112.24 ± 54.46 mg/dL and 168.73 ± 37.59 to 166.27 ± 41.54 mg/dL, respectively). The LDL:HDL ratio also improved insignificantly from 3.06 ± 0.87 (at baseline) to 3.02 ± 0.86 after 12 weeks of DRB intervention. At week 12, there were no significant differences in FBG, insulin, HOMA-IR, and QUICKI between the DRB and placebo groups (96.57 ± 22.40 vs. 101.19 ± 31.79 mg/dL, 8.38 ± 3.88 vs. 8.99 ± 5.04 uIU/mL, 2.15 ± 1.53 vs. 2.10 ± 1.29 and 0.35 ± 0.03 vs. 0.35 ± 0.04, respectively). However, HbA1c level significantly decreased by − 3.59% (5.89% ± 0.76% to 5.66% ± 0.62%, *p* = *0.0001*) in participants supplemented with DRB. In addition, the effect of DRB on lowering HbA1c levels was observed as early as week 6 (Table 3). Additionally, there was no significant difference in the hs-CRP concentration between the control and DRB groups after the 12-week intervention (2.79 ± 2.35 vs. 2.09 ± 1.94 mg/L, respectively). In addition, hs-CRP concentrations in participants of the DRB group at week 12 were insignificantly different when compared to those at baseline (1.88 ± 1.59 to 2.09 ± 1.94, *p* = *0.0970*). Similarly, the concentration of homocysteine was not significantly different when compared between the control and DRB groups at baseline (10.69 ± 3.07 μmol/L vs. 11.37 ± 3.29 μmol/L) and after 12 weeks of intervention (11.06 ± 2.46 μmol/L vs. 10.98 ± 3.20 μmol/L) (Table 3).

### Dietary intake parameters

The average energy intake in the DRB group decreased significantly from baseline to the end of the study (1,770.4 ± 257.16 vs. 1,646.16 ± 339.87 kcal/d., *p* = *0.0120*). In addition, participants in the DRB group reported a lower consumption of carbohydrates and fat (225.37 ± 45.77 g/day and 64.80 ± 16.39 g/day at baseline to 216.55 ± 51.27 g/day and 59.62 ± 21.88 g/day, respectively). Additionally, supplementation of DRB significantly increased the mean dietary fiber intake from 9.48 ± 5.35 g/day at baseline to 15.38 ± 3.33 g/day after the 12-week intervention (*p* < *0.0001*) (Table 3).

### Gastrointestinal symptom parameters

**Fig. 2:**
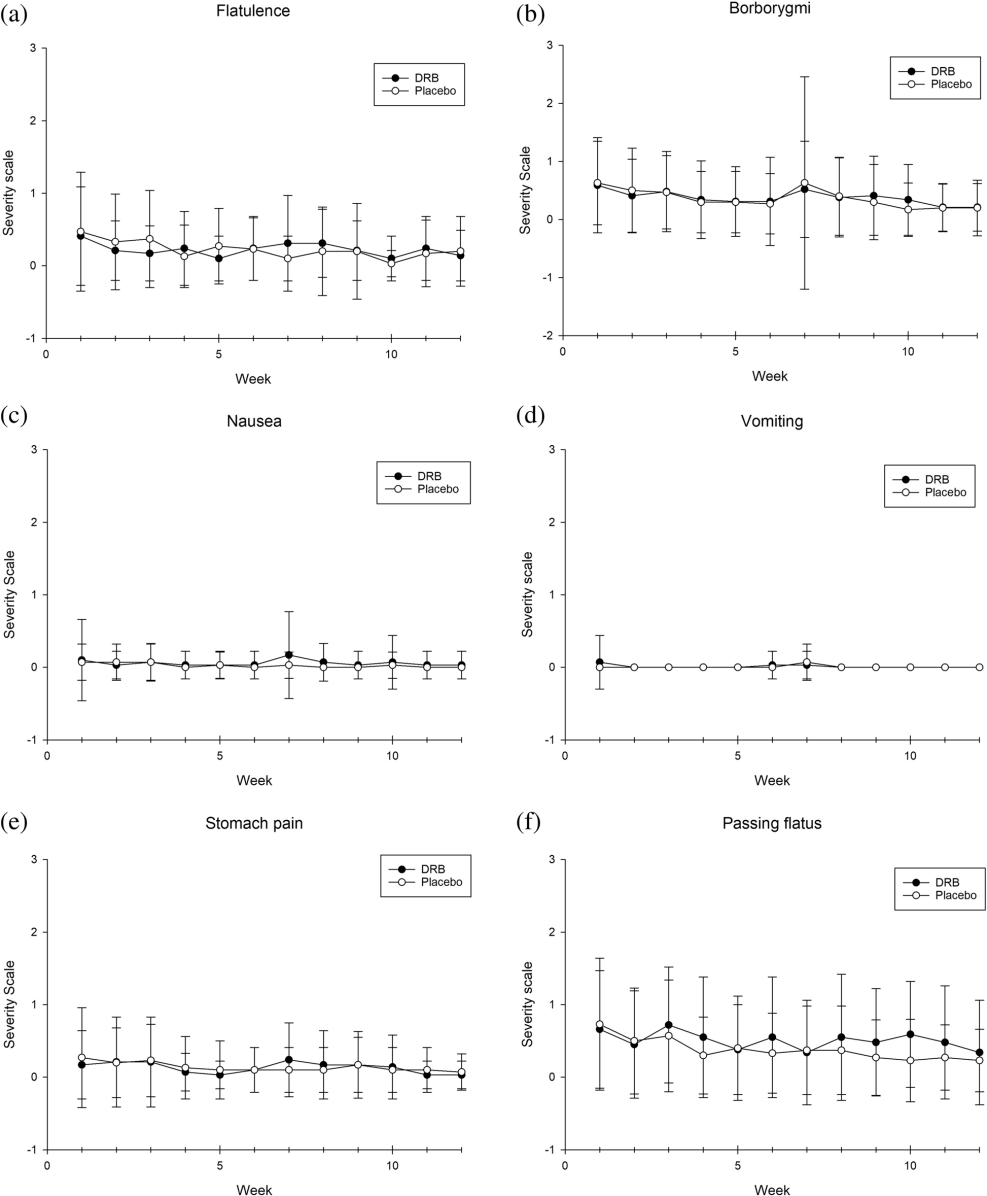
Gastrointestinal symptoms: **a** Flatulence, **b** Borborygmi, **c** Nausea, **d** Vomiting, **e** Stomach pain, and **f** Passing flatus. Mean ± SD of self-reported gastrointestinal symptoms by participants at each follow-up week. x-axis = week of intervention, y-axis = intensity of symptoms, scored as 0 (none), 1 (mild), 2 (moderate), or 3 (severe)

**Fig. 3:**
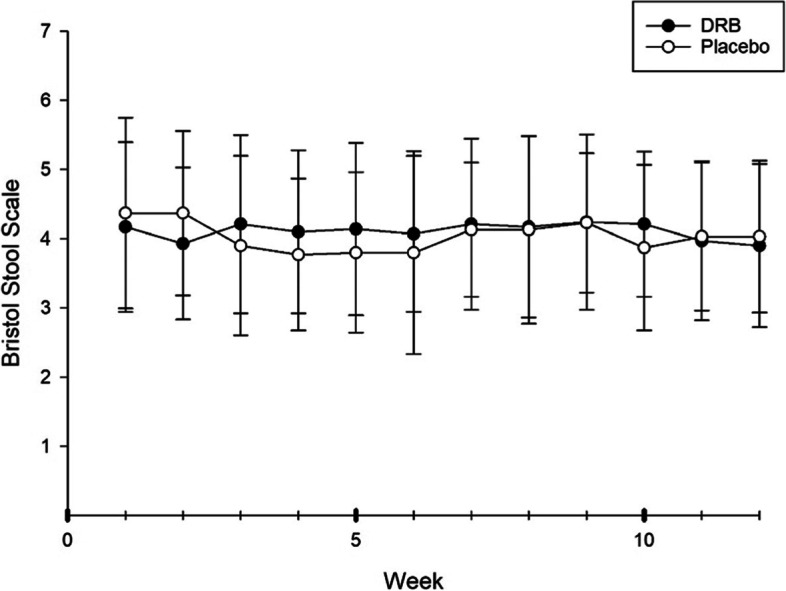
Bristol stool scale. Mean ± SD of classification of stool by the Bristol stool scale by participants at each follow-up week. x-axis = week of intervention, y-axis = type of stool, type 1 = separate hard lumps, type 7 = watery, no solid pieces

The result showed that 96.55% of participants in the DRB group reported no gastrointestinal symptoms after supplementation, while 3.45% reported mild gastrointestinal symptoms, including flatulence, borborygmi, nausea, stomach pain, and passing flatus (Fig. 2). Participants in the DRB group reported an improvement in the prevalence of a healthy stool form (type 4 stool form) from 34.48% at baseline to 48.28%, while there was no change in the prevalence of the type 4 stool form in the control group (35.48–32.26%) (Fig. 3).

## Discussion

The present study reported that 30 g of DRB supplementation daily for 12 weeks does not significantly alter body weight and other body composition indices, in accordance with a systematic review of randomized controlled trials, which reported that fiber consumption had an insignificant effect on energy intake and body weight [24]. Even though it has been reported that soluble fiber reduces appetite and increases satiety, the limited amount of soluble fiber (6.16% [w/w]) contained in DRB may provide an explanation for these null outcomes.

Daily DRB supplementation effectively reduced both systolic and diastolic blood pressure in overweight and obese adults with hypercholesterolemia. A previous *in vitro* study showed that rice bran peptide hydrolysate of molecular size > 50 and 10–50 kDa could inhibit angiotension-1 converting enzyme (ACE) by 78 and 55%, respectively [25]. The plausible mechanism of the effect of rice bran protein on blood pressure includes ACE inhibitory activity, enhancement of the eNOS pathway, an increase in NO bioavailability, and the attenuation of ROS formation through inhibition of the NADPH oxidase system [10, 11]. The three peptides, Leu-Arg-Ala, contained in rice bran have been demonstrated to induce vasorelaxation mediated by the NO pathway in the endothelium of blood vessels [26].

This study demonstrated that DRB supplementation reduced HbA1c concentrations by 3.59%. There are various possible mechanisms for this improvement, including enhanced secretion of glucose-dependent insulinotropic polypeptide (GIP) [27], reduced appetite and food intake [28], and inhibition of GLUT 4 transporters [29]. In addition, it was well established that insoluble fiber may increase fecal bulk and decrease intestinal transit time, thus resulting in decreased absorption of glucose and other simple carbohydrates and an 8% improvement in insulin sensitivity [30].

In this study, DRB supplementation had an insignificant effect on FBG, serum insulin, HOMA-IR, and QUICKI. Even though previous studies have demonstrated a reduction in FBG after DRB supplementation, most them were conducted on patients with Type I or II diabetes mellitus [31, 32]. The normoglycemic status at baseline and tightly control glucose homeostasis in healthy young adults in this study may be partly responsible for these null effects.

Nevertheless, cholesterol-lowering properties were reported in a full-fat rice bran supplementation study. This study observed insignificant reduction of TC, TG, and LDL-c concentrations by 3.12% ± 9.47%, 1.32% ± 24.86%, and 1.53% ± 10.90%, respectively, after DRB supplementation. This null effect might be because of limited amounts of unsaponifiable compounds (γ-oryzanol, β-sitosterol, and tocotrienols) contained in DRB. These unsaponifiable compounds have been reported to responsible for the cholesterol-lowering properties of DRB [32]. Since these compounds have similar structures to that of cholesterol, they may compete with cholesterol absorption in the small intestine [33]. Furthermore, β- and γ-tocotrienols can inhibit 3-hydroxy-3-methyl-glutaryl-coenzyme A (HMG-CoA) reductase, thus reducing endogenous cholesterol synthesis [34]. During the oil extraction, unsaponifiable compounds were excluded to some extent. With the limited amount of these compounds, DRB may not effectively improve blood lipid profiles. This study, therefore, only observed a trend toward a cholesterol-lowering effect of DRB supplementation.

Mean reductions in daily energy intake (120 kcal) and dietary fat were observed, whereas carbohydrate and protein consumption remained constant. This effect might be a consequence of an increase in dietary fiber consumption of 7.78 g (7.27 g insoluble and 0.51 g soluble). It has been proven that insoluble fiber can reduce appetite and increase fat satiety, with a consequent decrease in caloric and fat intake [28].

This study demonstrated a null effect of DRB on hs-CRP, anti-inflammatory cytokines, and homocysteine. Previous studies showed that phytochemicals and unsaponifiable compounds exhibit potent free-radical scavenging activity [35, 36]. It was also reported that rice bran polysaccharide increased antioxidant enzyme activity in mice while decreasing the MDA content [37]. With limited amounts of these beneficial compounds and their components after oil extraction of rice bran, DRB posed an insignificant effect on hs-CRP concentrations. In addition, the amounts of vitamin B6 in 30 g of DRB may not have been sufficient to significantly lower homocysteine levels. Additionally, the amount of vitamin B6 in the 30 g DRB may not be an exclusive solution for improving homocysteine levels.

The present study reported that DRB supplementation does not cause gastrointestinal disturbance. However, an improvement in the prevalence of a healthy stool form was reported. As mentioned previously, DRB contains mainly insoluble fiber, which produces the stool bulk effect and reduces intestinal transit time [38–40]. Additionally, another study by Tomlin and Read showed that rice bran increased stool mass and stool frequency after 10 days of supplementation. They also suggested that the stool bulking effect of rice bran is caused by a high content of insoluble fiber [41].

The present study used a randomized controlled trial to minimize bias. It also provided information about the effects of DRB supplementation on anthropometrics, blood biochemical parameters, and dietary intake in overweight/obese adults with hypercholesterolemia. The results of this study will benefit the food manufacturing sector by providing information on using DRB as an active ingredient in functional foods. However, the study had some limitations. First, it did not measure physical activity, a significant confounding factor, during the intervention period. Second, spontaneous improvement with a placebo in a randomized design without cross-over was another major limitation of this study. Third, it recruited otherwise healthy overweight/obese adults with hypercholesterolemia; therefore, this result cannot be generalized to other populations, and so it cannot apply to any diabetes mellitus patients. Further studies related to the mechanism of the metabolic effects of DRB are necessary to describe a clear picture of DRB and its potential use in the industrial sector.

## Conclusions

DRB could be incorporated as a functional food ingredient to significantly improve blood pressure. It improves HbA1c levels and lowers calorie and fat intake. On the other hand, DRB has no significant effect on lowering blood cholesterol levels. Further study is needed to evaluate the mechanisms of DRB supplementation on these beneficial metabolic changes.

## Supplementary Information

**Additional file 1.** CONSORT Checklist.

**Additional file 2.** Gastrointestinal symptoms evaluation.

## Data Availability

The datasets used and/or analyzed during the current study are available from the corresponding author on reasonable request.

## Abbreviations

ACE: Angiotensin-converting enzyme
BMI: Body mass index
CLIA: Chemiluminescence immunoassay method
DRB: Defatted rice bran
FBG: Fasting blood glucose
GIP: Glucose-dependent insulinotropic polypeptide
HMG-CoA: 3-Hydroxy-3-methyl-glutaryl-coenzyme A
HOMA-IR: Homeostatic model assessment of insulin resistance
NO: Nitric oxide
QUICKI: Quantitative insulin sensitivity check index
RFM: Relative fat mass
RPH: Rice protein hydrolysate
TC: Fasting total cholesterol
TG: Triglycerides
VAI: Visceral adiposity index
