# Supplementary material for: Effect of defatted rice bran supplementation on metabolic parameters and inflammatory status in overweight/obese adults with hypercholesterolemia: a randomized, placebo-controlled intervention
Source: BMC Nutr. 2022 Sep 1;8:94. doi: 10.1186/s40795-022-00586-9 (PMC9434873; doi:10.1186/s40795-022-00586-9)
Supplement: Supplementary file 2 — Additional file 2. Gastrointestinal symptoms evaluation. [file 40795_2022_586_MOESM2_ESM.docx]

**GASTROINTESTINAL SYMPTOMS EVALUATION**

Date/ day________________________________________________

Instruction : If you feel pain following the product administration, please rate the symptoms by putting a mark (√) on the listed table below. If you do not feel anything, please put a mark (√) on “none”.

| **Symptoms** | **None** | **Mild** | **Moderate** | **Severe** |
| --- | --- | --- | --- | --- |
| Abdominal pain |  |  |  |  |
| Borborygmi |  |  |  |  |
| Nausea |  |  |  |  |
| Vomiting |  |  |  |  |
| Bloating |  |  |  |  |
| Flatulence |  |  |  |  |

Comments: ________________________________________________________________________________________________________________________________________________________________________________________________________________________________

**BRISTOL STOOL CHART**

Date/ day________________________________________________

Please tick one of the types of the stool form which closely describe your stool form

| **Type** | **Description** | **Image** |
| --- | --- | --- |
| Type 1 | Separate hard lumps, like nuts | 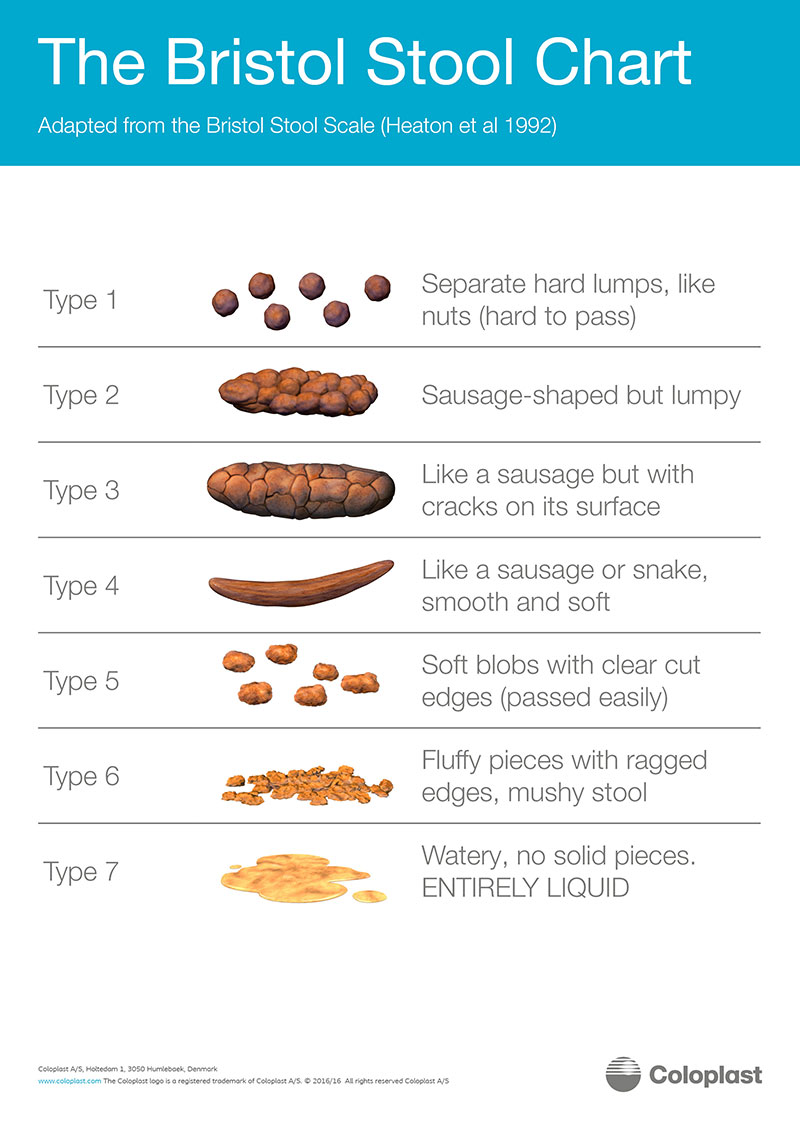 |
| Type 2 | Sausage-shaped but lumpy | 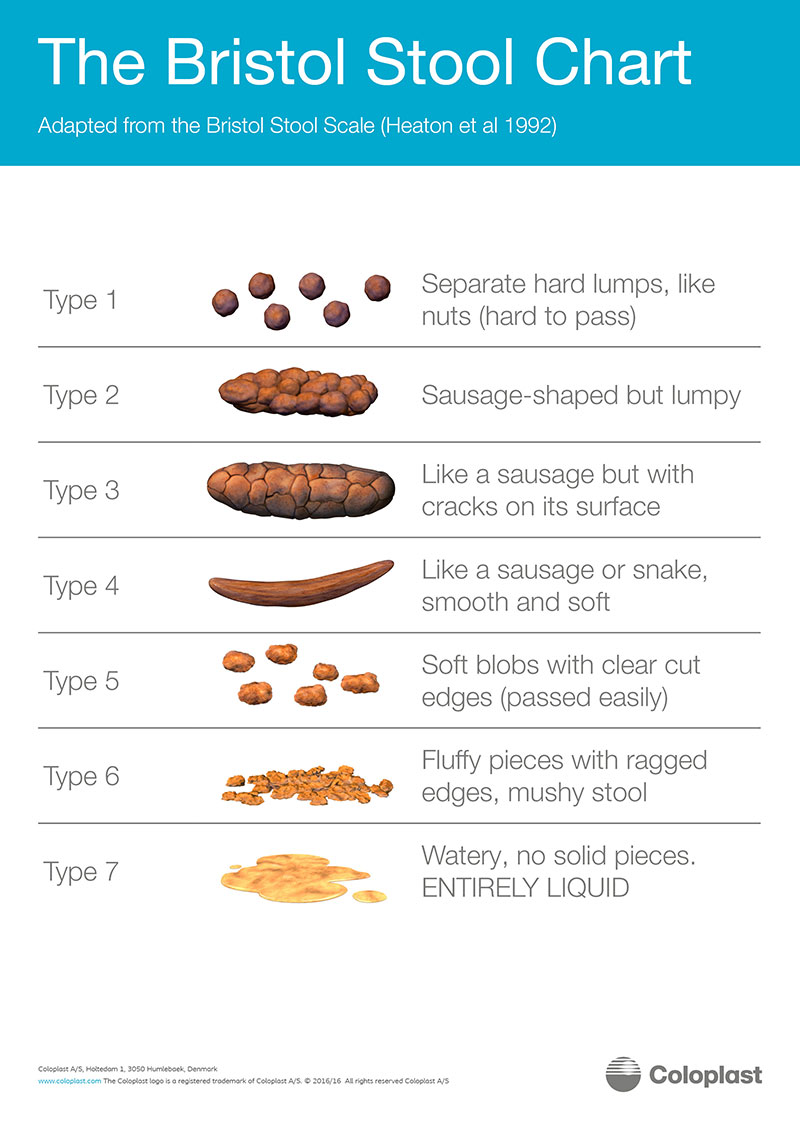 |
| Type 3 | Like sausage or snake but with cracks on its surface | 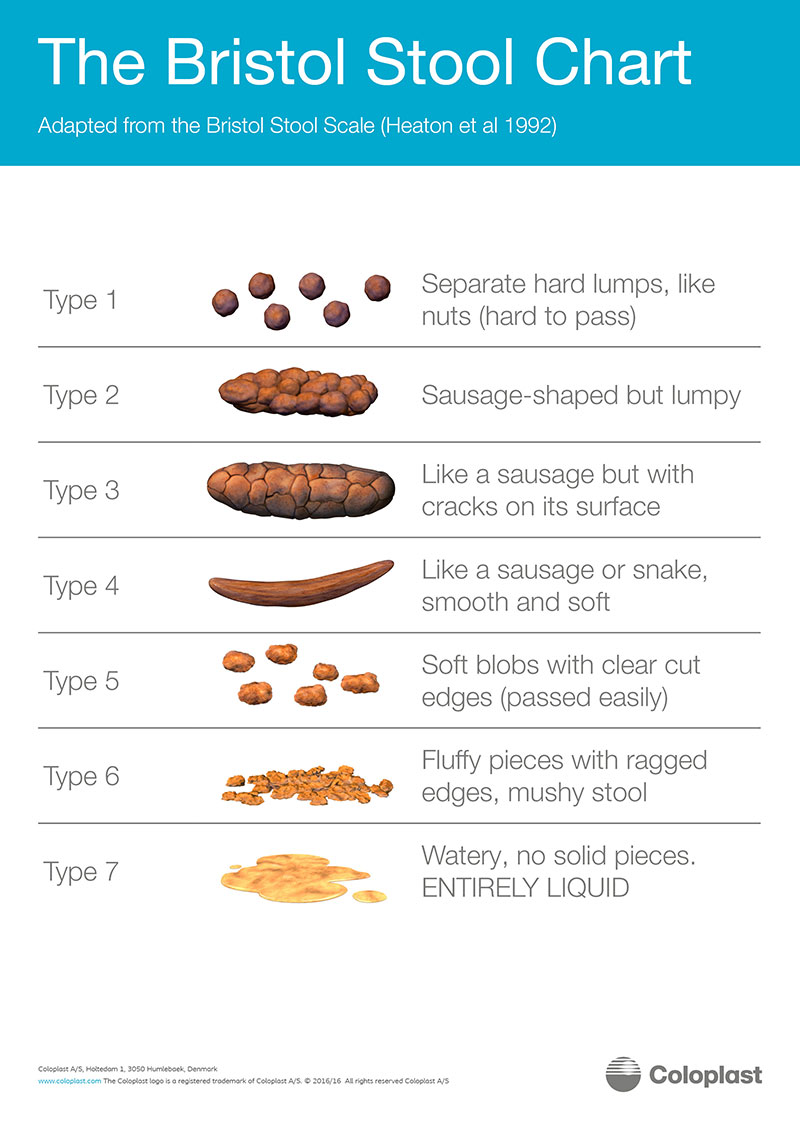 |
| Type 4 | Like sausage or snake, smooth and soft | 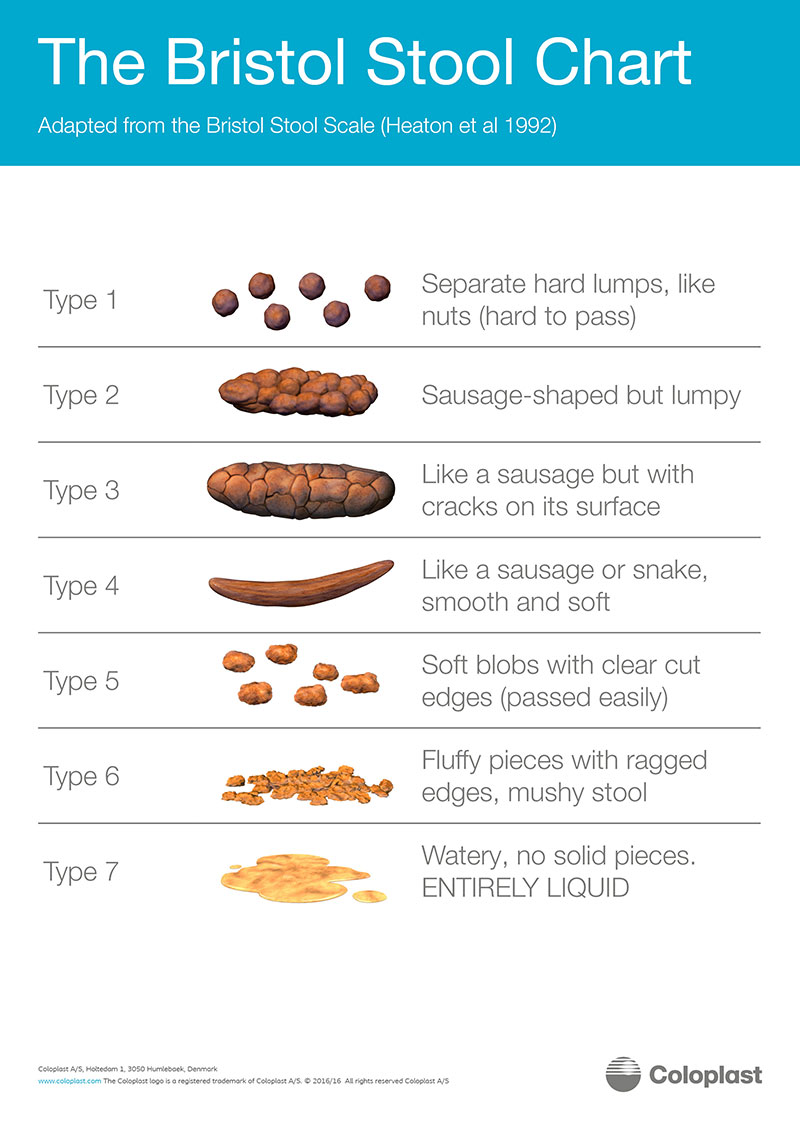 |
| Type 5 | Soft blobs with clear-cut edges | 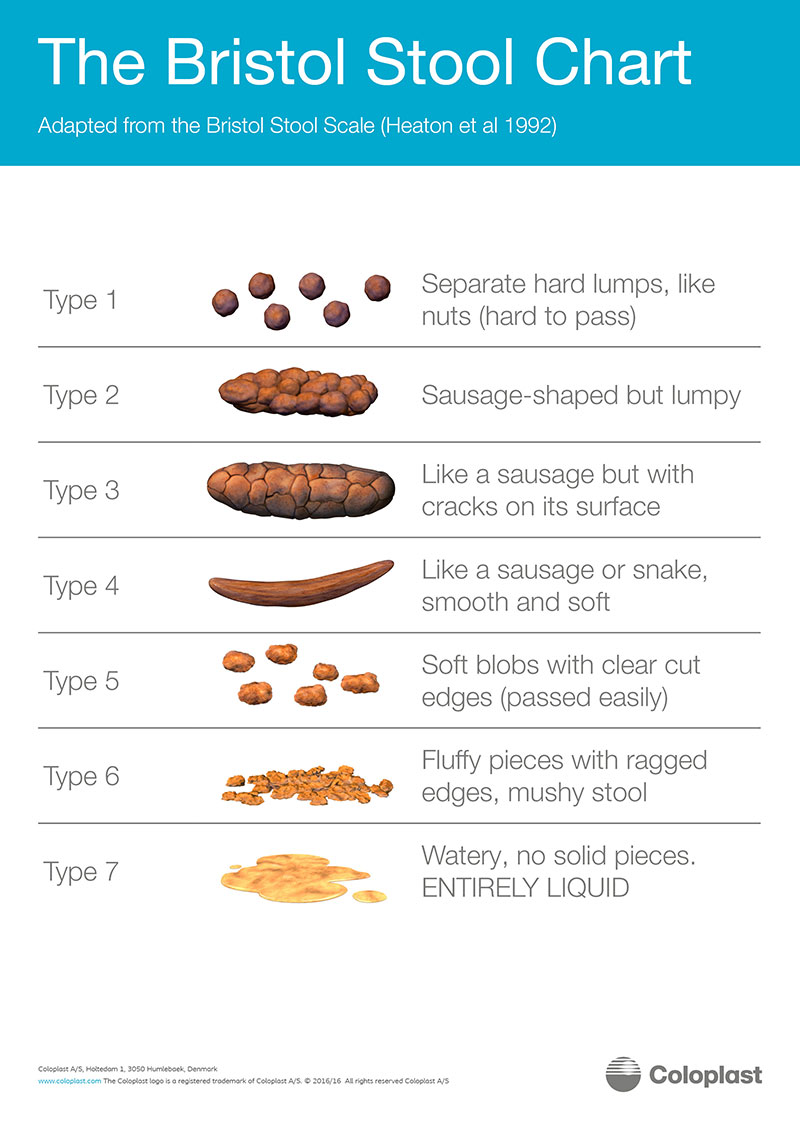 |
| Type 6 | Fluffy pieces with ragged edges, a mushy tool | 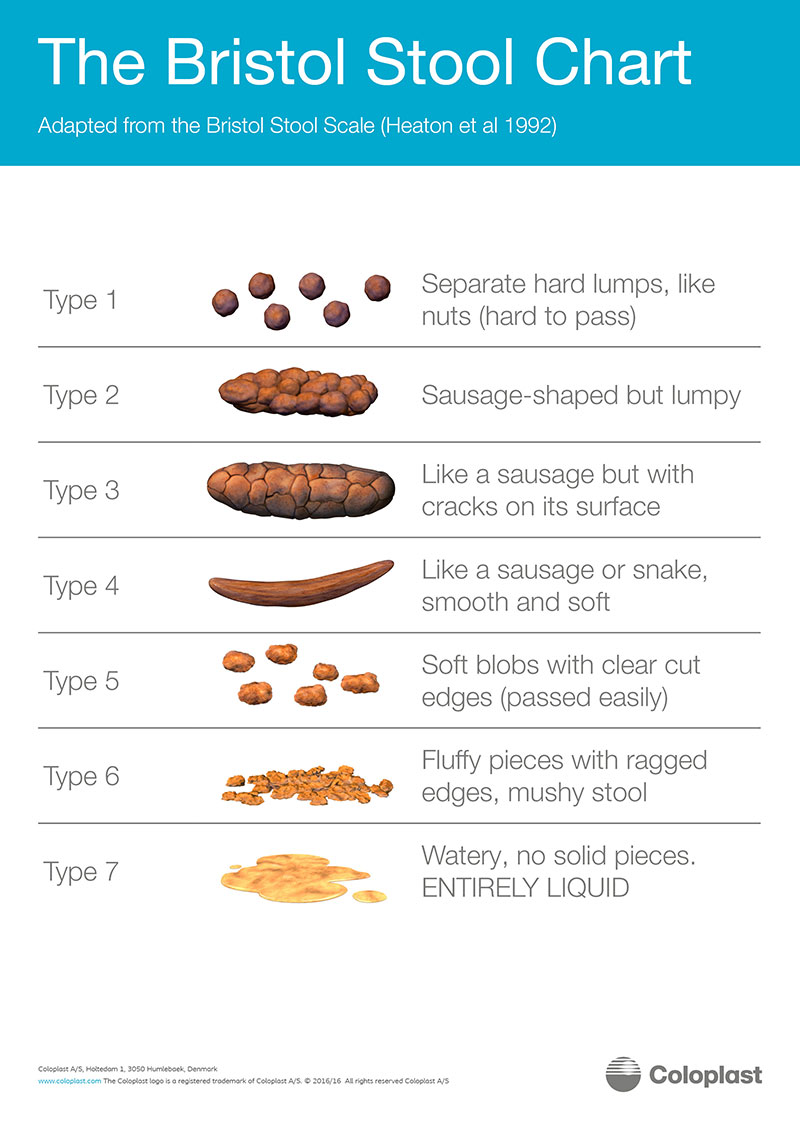 |
| Type 7 | Watery, no solid pieces | 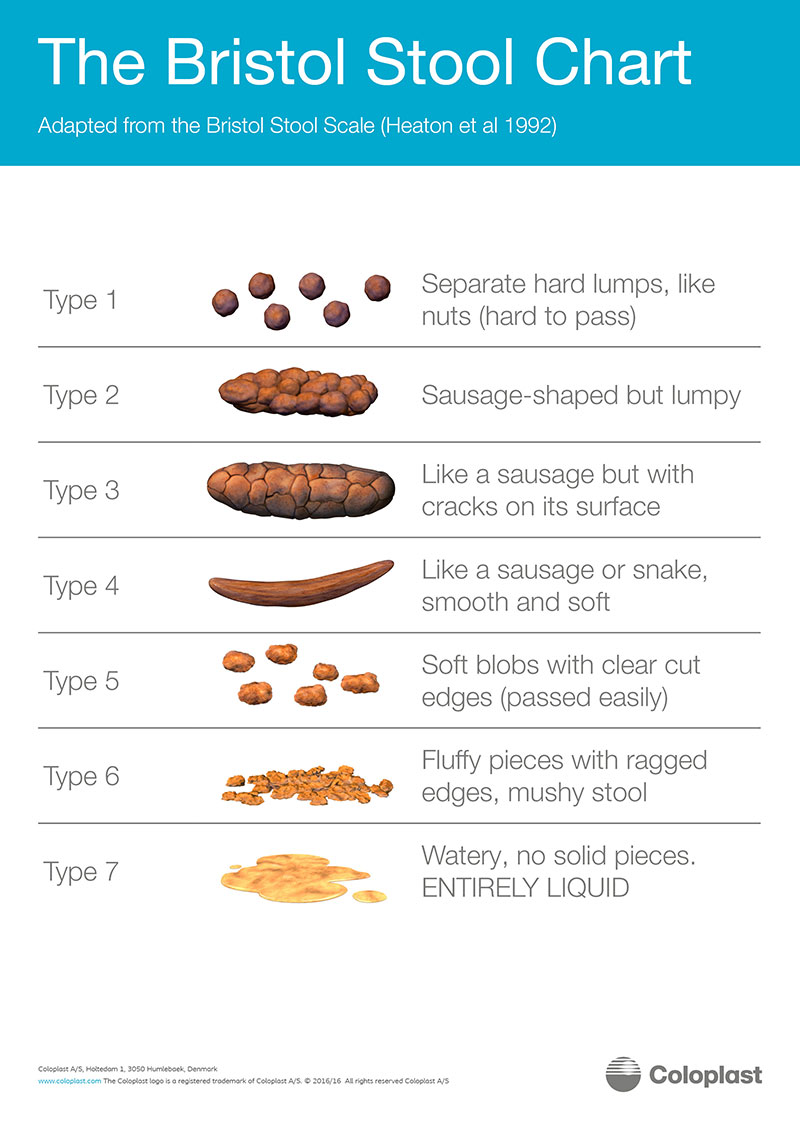 |

Comments (Time of the day, Amount, other symptoms): ________________________________________________________________________________________________________________________________________________________________________________________________________________________________

Instruction for booklet recording and lifestyle during participate the research

1. Dietary record: please record your daily food intake 3 days/week, 2 weekdays and 1 weekend day. Choose any days in the week as you desire. The dietary record should include date, list of foods and beverages, and food ingredients.
2. Gastrointestinal symptoms evaluation: please record your gastrointestinal symptoms 1 day/week. If any unusual symptom occurs, note the frequency of that symptom.
3. Bristol stool chart: please record your stool form 1 day/week. If any unusual symptom occurs, note the frequency of that symptom.
4. Please maintain your physical activity, exercise, and keep your food intake unchanged during the intervention period.
5. Refrain from drinking alcoholic beverages and smoking during the intervention period.
6. Refrain from using any food supplements during intervention period.
